# Ocular Toxicity Secondary to *Asclepias physocarpa*: The Balloon Plant

**DOI:** 10.1155/2014/829469

**Published:** 2014-07-02

**Authors:** Susana Pina, Catarina Pedrosa, Cristina Santos, Bernardo Feijóo, Peter Pego, Cristina Vendrell, Maria João Santos, Isabel Prieto

**Affiliations:** Ophthalmology Department, Hospital Prof. Dr. Fernando da Fonseca, Estrada IC-19, 2720-276 Amadora, Portugal

## Abstract

We report a case of a 65-year-old woman with symptoms of blurred vision and ocular irritation a few hours after accidental contact of the right eye with *Asclepias physocarpa* milky latex. Observation showed a diffuse conjunctival hyperemia and stromal corneal edema with Descemet's membrane folds. Recovery was fast and apparently complete in less than one month. However, specular microscopy at 6-months follow-up showed an abnormal endothelial morphology as sequelae, suggesting this condition is not as innocuous as it has been suggested.

## 1. Introduction

Plants of the genus* Asclepias* originate from regions of the tropics and subtropics [1, 2]. Often referred to as milkweeds for their milky latex, they grow as shrubs up to 1.5 m tall and have green stems covered with velvety hair [3].* Asclepias physocarpa* is a species commonly known as balloon plant due to its characteristic swelling balloon-like pods which are full of seeds. Their white flowers are very attractive to butterflies. Due to their decorative features, they are artificially cultivated in different parts of the globe.* Asclepias* milky latex is considered toxic because it contains cardenolides (cardiac glycosides). These natural toxins are intended to protect plants and insects, mainly butterflies, from predation and are well known causes of poisoning and death of livestock [1–4]. Despite their toxicity, their cardiotonic activity is the basis of many of their pharmacological uses [4]. Their mechanism of action consists in the ability to bind and inhibit the membrane protein Na^+^/K^+^-ATPase present in almost all cellular membranes, including the corneal endothelium [1, 2, 4]. We report a clinical observation of ocular toxicity after exposure to* Asclepias physocarpa* latex.

## 2. Case Presentation

**Figure 1:**
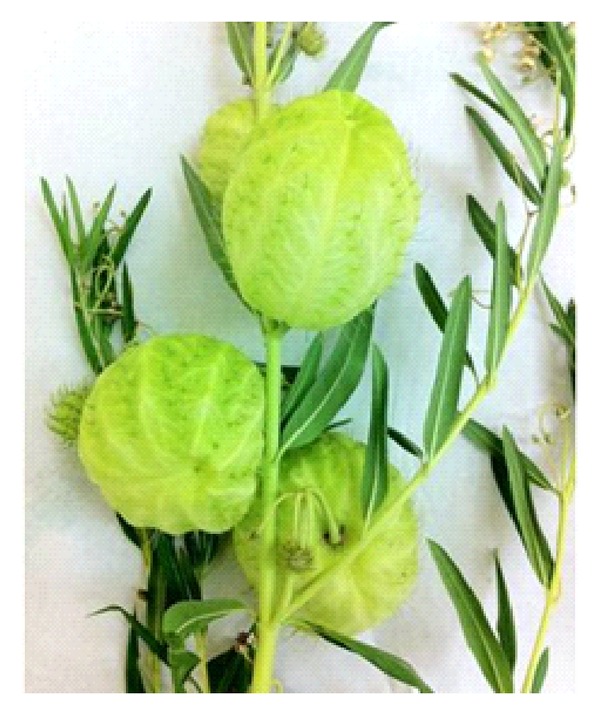
Specimen of* Asclepias physocarpa* brought by the patient.

A 65-year-old woman, with controlled systemic hypertension and history of cataract surgery to the left eye, florist by profession, presented to our clinic with symptoms of blurred vision and ocular irritation 6 hours after accidental contact of the right eye (RE) with* Asclepias physocarpa* milky latex (Figure 1).

**Figure 2:**
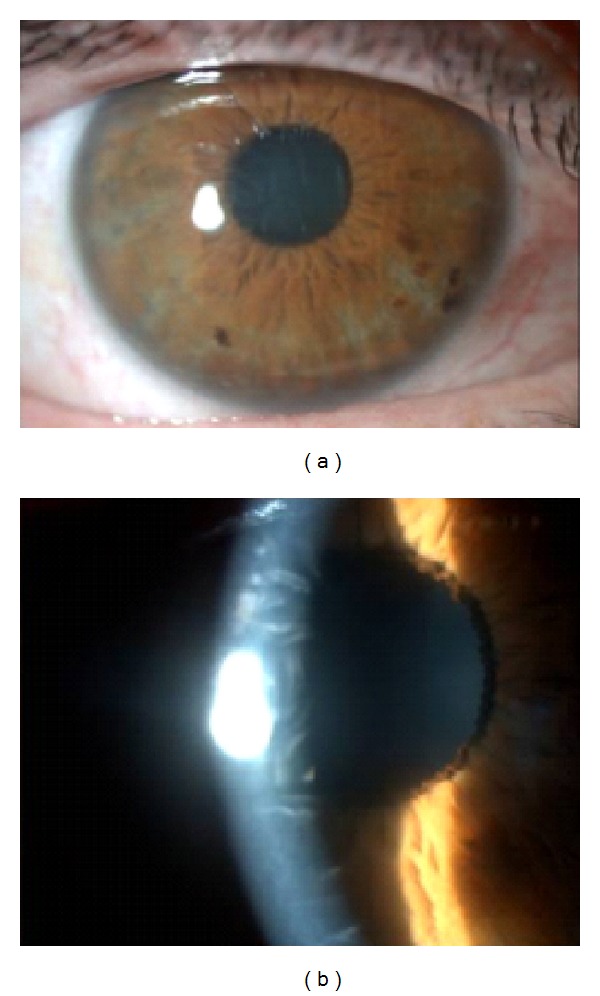
RE: diffuse conjunctival hyperemia, intact corneal epithelium, and corneal swelling with Descemet's membrane folds.

Clinical examination showed a best corrected visual acuity (BCVA) of 20/80 in the RE and 20/20 in the left eye (LE). Biomicroscopy of the RE showed diffuse conjunctival hyperemia and exuberant stromal corneal edema with Descemet's membrane folds, without corneal epithelium injury or ocular discharge (Figure 2). Anterior chamber was quiet. Examination of the LE was unremarkable, except for pseudophakia. Intraocular pressure was within the normal range in both eyes (13 mmHg in the RE and 14 mmHg in the LE).

**Figure 3:**
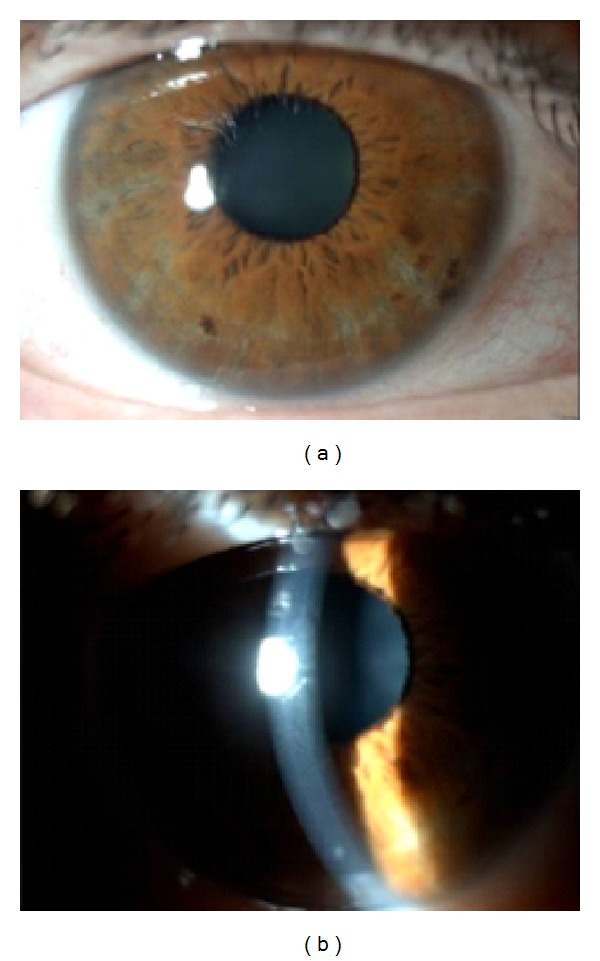
RE: residual corneal edema and softer Descemet's folds.

After flushing with saline solution, supportive treatment with topical dexamethasone 0.1% (b.i.d), ofloxacin 0.3% (t.i.d), hypertonic NaCl solution 5% (b.i.d), and artificial tears was applied. After 48 h, a significant improvement in signs and symptoms of the RE was observed (Figure 3) with BCVA reaching 20/25.

**Figure 4:**
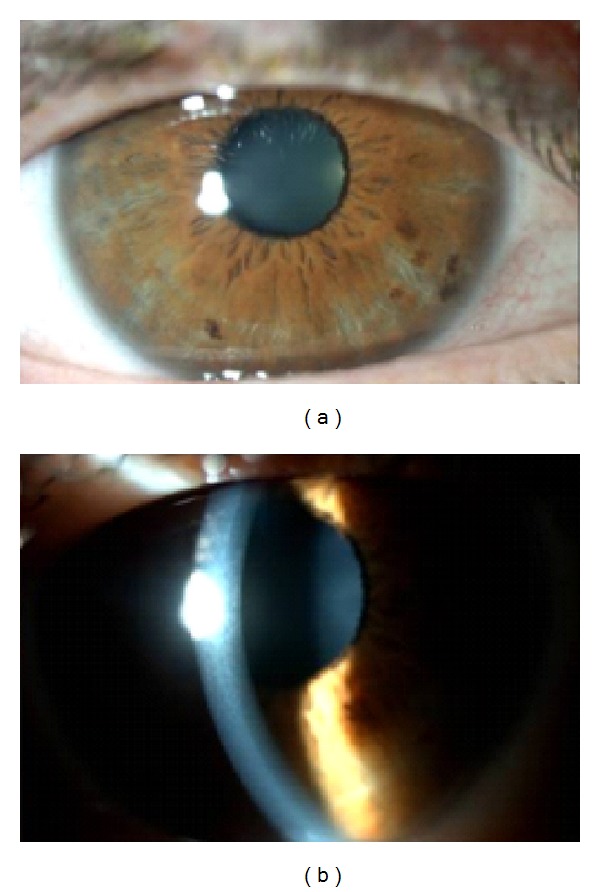
RE: almost complete resolution of the edema.

One week after presentation, almost complete recovery of the RE was achieved, with resolution of the corneal edema and only faint folds in Descemet's membrane (Figure 4) with a BCVA of 20/20. Treatment was suspended after one week due to almost complete resolution of the edema.

**Figure 5:**
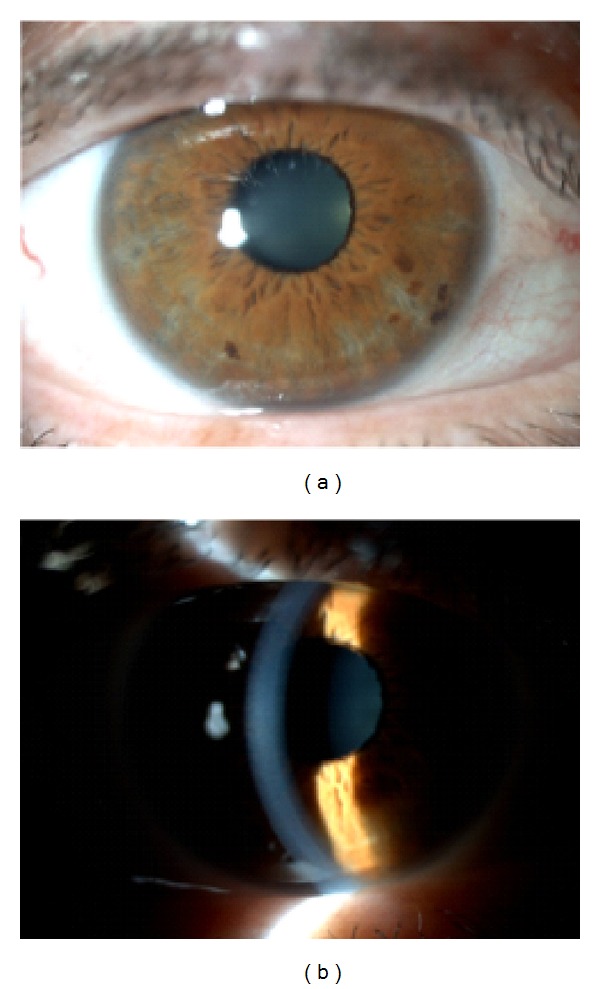
RE: complete resolution at biomicroscopy.

At one- and six-month follow-up, no clinical signs were evident on slit-lamp examination, with complete resolution of the edema (Figure 5) and vision remained stable at 20/20.

**Figure 6:**
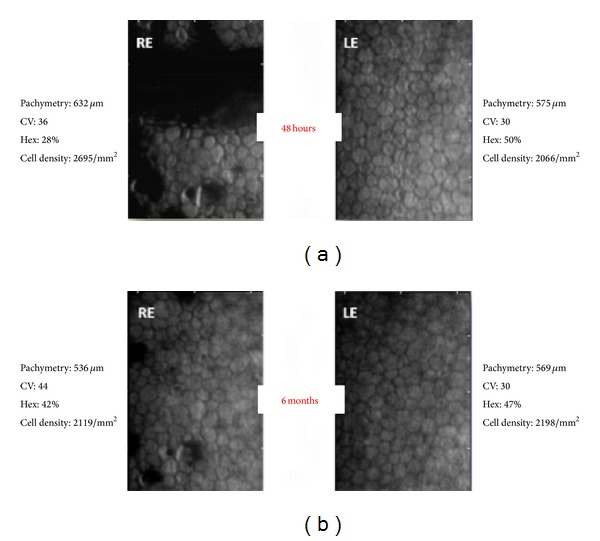
Specular microscopy of the RE shows polymegathism and pleomorphism; CV: coefficient of variation; Hex: percentage of hexagonal cells.

Despite this apparently resolved clinical situation, specular microscopy of the RE obtained during the course of disease showed an altered endothelial cell pattern (Figure 6).

## 3. Discussion

*Asclepias* are known for containing toxic cardiac glycosides in their latex composition. These compounds inhibit the Na^+^/K^+^-ATPase [1, 2, 4] which is the primary protein responsible for corneal hydration, being expressed in the basolateral membrane of corneal endothelial cells. When in contact with the eye, cardenolides have the ability to fully penetrate through the intact cornea and inhibit the endothelial sodium-potassium pump causing edema, with loss of the corneal normal transparency [1, 5].

According to the literature, the use of topical steroids may increase the activity of the sodium-potassium pumps that remained functional, accelerating recovery. Moreover, corneal edema may also resolve spontaneously in a few days, when inhibition by cardenolides has ceased [1, 6].

A few hours after accidental eye contact with* Asclepias*' sap, our patient presented with conjunctival hyperemia and stromal corneal edema, with Descemet's folds and an intact epithelium. Clinical evolution, with supportive treatment including topical steroids, showed a considerable improvement in 48 hours. Treatment was suspended after one week due to almost complete resolution of the edema. Prolonged therapy with topical steroids does not seem to be beneficial since spontaneous corneal edema resolution occurs. However, it does seem to hasten the resolution of the edema.

Complete visual recovery and corneal edema resolution were achieved within a few weeks. The two similar published cases report an analogous clinical course with full recovery and without sequelae [1, 2]. However, despite being apparently a self-limited situation, the modification of the endothelial cells pattern in our patient's RE suggests the existence of a residual effect.

After 48 h of presentation, in addition to endothelial folds and excrescences, an increased pachymetry (632 *μ*m) was observed, which is compatible with the residual corneal edema. The observed excrescences may represent an alteration in the metabolism of endothelial cells following the sustained aggression. The other assessed parameters—coefficient of variation of 36%, 28% hexagonal cells, and cell density of 2,695 cells/mm^2^—are not reliable given the large distortion of the endothelium at the time of observation.

At the 6-month follow-up, specular microscopy of the RE showed a normal pachymetry of 536 *μ*m, demonstrating a complete resolution of the edema. However, the elevated coefficient of variation (44%) and the decreased percentage of hexagonal cells (42%) indicate polymegathism and pleomorphism, both of which are signs of endothelial distress [7]. Moreover, compared to the LE, the coefficient of variation was higher and the percentage of hexagonal cells was lower, confirming endothelial distress in the RE. Cell density of the RE (2,119 cells/mm^2^) was similar to the LE (2,198 cells/mm^2^), both within the normal range. However, the LE had undergone cataract surgery 3 years earlier, a surgical procedure that can decrease the number of endothelial cells [8], making the comparison of this parameter difficult and less reliable since we do not have the cell count prior to surgery. Nevertheless, we can speculate that the endothelial cell density of the RE prior to this episode was probably higher than in the LE and that the contact with* Asclepias*' sap brought down this count to values comparable to the LE postsurgery.

The finding of a permanent change in cell pattern on specular microscopy suggests an altered endothelial function with possible future repercussions, such as lower tolerance to an intraocular surgery or another eye insult.

We propose that possible exposures to plant sap should be included in the differential diagnosis of patients presenting with sudden corneal edema. Clinical history is crucial for the recognition of this clinical situation, which must be correctly diagnosed to allow an appropriate management. Simple health education basic rules such as washing hands, avoiding rubbing the eyes, using gloves, and eye protection when handling this kind of plants, so often used in gardens and for decorative purposes, may be sufficient to prevent this kind of injury.
